# Mechanistic insight into the efficient packaging of antigenomic S RNA into Rift Valley fever virus particles

**DOI:** 10.3389/fcimb.2023.1132757

**Published:** 2023-02-16

**Authors:** Breanna Tercero, Kaori Terasaki, Krishna Narayanan, Shinji Makino

**Affiliations:** ^1^ Department of Microbiology and Immunology, The University of Texas Medical Branch, Galveston, TX, United States; ^2^ Institute for Human Infections and Immunity, The University of Texas Medical Branch, Galveston, TX, United States; ^3^ Center for Biodefense and Emerging Infectious Diseases, The University of Texas Medical Branch, Galveston, TX, United States; ^4^ UTMB Center for Tropical Diseases, The University of Texas Medical Branch, Galveston, TX, United States; ^5^ The Sealy Institute for Vaccine Sciences, The University of Texas Medical Branch, Galveston, TX, United States

**Keywords:** Rift Valley fever virus, viral RNA packaging, CLIP-Seq analysis, bunyavirus, viral RNA-protein interaction, suppression of innate immune response

## Abstract

Rift Valley fever virus (RVFV), a bunyavirus, has a single-stranded, negative-sense tri-segmented RNA genome, consisting of L, M and S RNAs. An infectious virion carries two envelope glycoproteins, Gn and Gc, along with ribonucleoprotein complexes composed of encapsidated viral RNA segments. The antigenomic S RNA, which serves as the template of the mRNA encoding a nonstructural protein, NSs, an interferon antagonist, is also efficiently packaged into RVFV particles. An interaction between Gn and viral ribonucleoprotein complexes, including the direct binding of Gn to viral RNAs, drives viral RNA packaging into RVFV particles. To understand the mechanism of efficient antigenomic S RNA packaging in RVFV, we identified the regions in viral RNAs that directly interact with Gn by performing UV-crosslinking and immunoprecipitation of RVFV-infected cell lysates with anti-Gn antibody followed by high-throughput sequencing analysis (CLIP-seq analysis). Our data suggested the presence of multiple Gn-binding sites in RVFV RNAs, including a prominent Gn-binding site within the 3’ noncoding region of the antigenomic S RNA. We found that the efficient packaging of antigenomic S RNA was abrogated in a RVFV mutant lacking a part of this prominent Gn-binding site within the 3’ noncoding region. Also, the mutant RVFV, but not the parental RVFV, triggered the early induction of interferon-β mRNA expression after infection. These data suggest that the direct binding of Gn to the RNA element within the 3’ noncoding region of the antigenomic S RNA promoted the efficient packaging of antigenomic S RNA into virions. Furthermore, the efficient packaging of antigenomic S RNA into RVFV particles, driven by the RNA element, facilitated the synthesis of viral mRNA encoding NSs immediately after infection, resulting in the suppression of interferon-β mRNA expression.

## Introduction

Rift Valley fever virus (RVFV) is transmitted by mosquitoes and is endemic in sub-Saharan Africa and several countries in the Middle East Asia. Human infection occurs from the bite of infected mosquitos or from direct transmission of the virus from infected animal tissues or blood. Human disease manifestations include transient incapacitating febrile illness (Francis and Magill, 1935; Sabin and Blumberg, 1947), encephalitis (Laughlin et al., 1978; Alrajhi et al., 2004), vision loss (Siam and Meegan, 1980; Siam et al., 1980; Deutman and Klomp, 1981; Al-Hazmi et al., 2005) and hemorrhagic fever (Abdel-Wahab et al., 1978; Yassin, 1978; Peters and Meegan, 1989; Peters and LeDuc, 1999; Balkhy and Memish, 2003). RVFV has the potential to spread to any area of the world (Peters and Meegan, 1989; Peters and LeDuc, 1999; Balkhy and Memish, 2003; Andriamandimby et al., 2010; Nguku et al., 2010; Hassan et al., 2011; Aradaib et al., 2013; Himeidan et al., 2014; Baba et al., 2016), including North America, by naturally occurring mosquito populations (Gargan et al., 1988). RVFV has also been considered a potentially exploitable agent for bioterrorism (Peters and Meegan, 1989; Peters and LeDuc, 1999).

**Figure 1:**
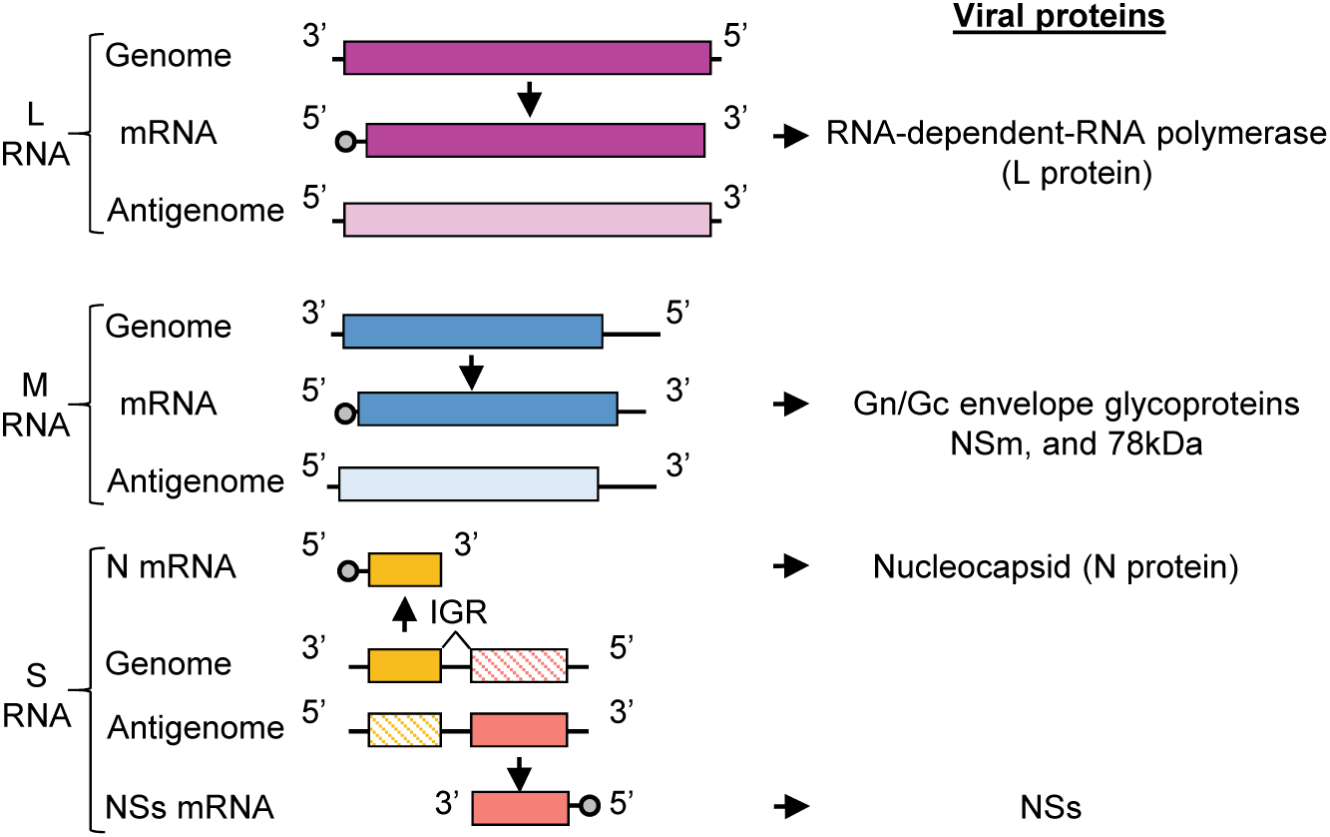
RVFV genome organization. RVFV RNA genome consisting of L, M, and S RNA segments. Genomic segments are negative sense and serve as templates for the generation of complementary antigenomic RNAs. The sizes of RVFV RNA segments are: L (6404 nt), M (3885 nt), S (1690 nt). The genomic strands of L and M segments are used to transcribe the L and M mRNAs, respectively. L mRNA encodes L protein and the M mRNA encodes the Gn/Gc glycoproteins, NSm, and 78kDa protein. The S segment utilizes an ambi-sense coding strategy wherein the N mRNA is transcribed from the genomic RNA and NSs mRNA is transcribed from the antigenomic RNA. N mRNA encodes for N protein and NSs mRNA encodes for the nonstructural protein, NSs. IGR, intergenic region.

RVFV belongs to genus *Phlebovirus*, family *Phenuiviridae*, order *Bunyavirales*, and is an enveloped, negative-sense RNA virus that has a tri-segmented RNA genome, consisting of L, M and S RNAs (**Figure 1**). The infectious virion consists of two envelope glycoproteins, Gn and Gc, and the three viral RNA segments (L, M, S) packaged in the form of ribonucleoprotein complexes (RNPs). The RNPs have a string-like appearance (Raymond et al., 2010), and are composed of L, M and S RNAs encapsidated by the nucleocapsid (N) protein and the RNA-dependent RNA polymerase (L) protein. L RNA encodes L protein, and M RNA encodes Gn and Gc proteins along with two accessory proteins, NSm and the 78-kDa protein (Terasaki et al., 2013; Terasaki et al., 2021). The S RNA uses an ambi-sense coding strategy for expression of N protein and nonstructural protein, NSs. N mRNA is transcribed from the genomic S RNA and translated to produce N protein, while NSs mRNA is transcribed from the antigenomic S RNA (complement sequence of genomic S RNA) and translated to produce NSs protein (Schmaljohn and Nichol, 2007). Both N and L proteins are essential for viral RNA synthesis (Dunn et al., 1995; Lopez et al., 1995; Blakqori et al., 2003; Ikegami et al., 2005a), which occurs in the cytoplasm. Virus assembly and budding occurs at the Golgi apparatus (Schmaljohn and Nichol, 2007).

The RVFV antigenomic RNAs carry the noncoding regions (NCRs) at their termini and the genomic and antigenomic S RNA carry an intergenic region between the N and NSs genes (**Figure 1**). The length of the 5’-end NCRs of the three antigenomic RNAs is short, ranging from 18 to 38 nucleotides (nt), whereas the length of the 3’-end NCRs of antigenomic RNAs are 110 nt, 271 nt and 34 nt for L RNA, M RNA and S RNA, respectively. The 3’-end NCRs of the antigenomic L and M RNAs include transcription termination sites, while the intergenic sites of the genomic S RNA and antigenomic S RNA carry the transcription termination sites of N mRNA and NSs mRNA, respectively (Ikegami et al., 2007) (**Figure 1**). Experiments using RVFV minigenomes showed that the presence of the 5’ NCR and the 3’ terminal 15 nt region of NCR of each antigenomic RNA segment is sufficient for minigenome RNA replication and their cognate genomic RNA packaging into virus-like particles (Murakami et al., 2012). Hereafter, we will refer to the 5’ NCR and the 3’ terminal 15 nt region of NCR of each antigenomic RNA and the corresponding regions of each genomic RNA as the minimal NCRs.

One of the fundamental and essential steps in a viral life cycle is the packaging of viral genome into virus particles for dissemination to new cells, organs and hosts. Many enveloped RNA viruses encode a matrix protein, which serves as a bridge between viral RNPs and viral envelope protein. However, all viruses in the *Bunyavirales* order and some members in the *Arenaviridae* family do not encode a matrix protein. It is hypothesized that the cytoplasmic tail of Gn functions as a matrix protein surrogate through its interaction with the viral RNPs and that this interaction drives the packaging of viral RNAs into those bunyaviruses lacking a matrix protein (Sherman et al., 2009; Piper et al., 2011; Strandin et al., 2013; Carnec et al., 2014; Spiegel et al., 2016; Guardado-Calvo and Rey, 2017). Studies using Crimean-Congo hemorrhagic fever orthonairovirus and hantavirus have shown the presence of a direct interaction between the Gn cytoplasmic tail and viral RNAs using an *in-vitro* assay (Estrada and De Guzman, 2011; Strandin et al., 2011). Also, for RVFV, it has been suggested that Gn protein directly interacts with viral RNAs (Piper et al., 2011; Carnec et al., 2014). Consistent with these studies and the hypothesis, our experiments using RVFV showed that: Gn binds to viral RNPs in infected cells (Tercero et al., 2021); Gn binds to all the components of viral RNPs, including L protein, N protein and viral RNA in infected cells (Tercero et al., 2021); and a positive correlation exists between a direct interaction of Gn with viral RNAs and the efficiency of RNA packaging into virus particles (Tercero et al., 2021). These studies established the importance of Gn-viral RNP interaction for RVFV RNA packaging, but the mechanism by which this interaction is initiated and established is unknown.

Infectious RVFV particles carry genomic L, M and S RNAs, and their antigenomic RNA counterparts are also packaged into RVFV particles (Ikegami et al., 2005b). Our past studies demonstrated that Gn binds to antigenomic S RNA substantially more efficiently than to antigenomic L and M RNAs, and antigenomic S RNA is efficiently packaged into viral particles (Tercero et al., 2021). The mechanism of the efficient interaction of Gn with antigenomic S RNA, among the antigenomic RNAs, is unknown. As the incoming RVFV RNPs trigger retinoic acid-inducible gene I (RIG-I)-mediated innate immune signaling (Weber et al., 2013), the efficient packaging of antigenomic S RNA, which facilitates the expression of NSs, an interferon (IFN) antagonist (Le May et al., 2004; Le May et al., 2008; Habjan et al., 2009; Ikegami et al., 2009; Kalveram et al., 2011; Kainulainen et al., 2014; Wuerth and Weber, 2016), immediately after infection (Ikegami et al., 2005b), would provide a functional advantage for establishing robust RVFV infection in IFN-competent mammalian hosts.

The present study investigated the mechanism of viral RNA packaging in RVFV by identifying the regions in viral RNAs that directly interact with Gn. Our data revealed the presence of multiple Gn-binding sites in the genomic and antigenomic viral RNAs. However, the minimal NCRs of the genomic RNAs lacked prominent Gn-binding sites. In contrast, the 3’ NCR of the antigenomic S RNA carried a prominent Gn-binding site. Analysis of a mutant RVFV lacking a part of this prominent Gn-binding site demonstrated the importance of this Gn-binding site for the efficient packaging of antigenomic S RNA. Also, this mutant virus, but not the parental RVFV, induced the early expression of IFN-β mRNA after infection. These data suggested that the direct binding of Gn to an RNA element in the 3’ NCR of the antigenomic S RNA promoted the efficient packaging of antigenomic S RNA into virions, which facilitated the synthesis of NSs mRNA immediately after infection, resulting in the suppression of IFN-β induction.

## Materials and methods

### Cells and viruses

Vero E6 cells were cultured in Dulbecco’s modified eagle medium, supplemented with 5% fetal bovine serum (FBS) and 1% penicillin-streptomycin. Huh7 cells were cultured in Dulbecco’s modified eagle medium supplemented with 10% FBS and 1% kanamycin (Nakabayashi et al., 1982). BSRT7/5 cells, which stably express T7 RNA polymerase, were cultured in Glasgow minimum essential medium (MEM) supplemented with 10% FBS, 10% tryptose phosphate broth (TPB), 1X MEM amino acid solution, and 1 mg/mL geneticin (Buchholz et al., 1999). MRC-5 cells were maintained in Eagle’s MEM containing 10% FBS, MEM Non-Essential Amino Acids Solution, and 1% sodium pyruvate.

All viruses used in the study were derived from MP-12 stain of RVFV and generated by an established reverse genetics system (Ikegami et al., 2006). RVFV-rLuc carries the *Renilla* luciferase (rLuc) open reading frame (ORF) in place of the NSs ORF in the S segment. The rescued viruses were amplified once in Vero E6 cells, titrated by plaque assay, and used for virus infections. For virus infection, cells were inoculated with virus for 1 h at 37˚C and then washed three times with phosphate buffered saline (PBS) before fresh medium was added.

### Rescue and amplification of RVFVΔ19

A recombinant PCR-based method was employed to generate the pProT7-S Δ19 construct, which expresses S Δ19 RNA carrying a 19nt deletion between nucleotide positions 1656 and 1675 in the 3’ NCR of antigenomic S RNA, by using the T7-driven plasmid pProT7-S encoding the antigenomic S RNA as a template. Sequencing of the S Δ19 plasmid construct confirmed the absence of unwanted mutations. The reverse genetics system to rescue RVFV S Δ19 was described as previously (Ikegami et al., 2006). Briefly, BSRT7/5 cells were co-transfected with T7-driven plasmids encoding L and N proteins, a pCAGGS-G plasmid encoding Gn/Gc proteins, and T7-driven viral RNA expression plasmids encoding L RNA, M RNA, and S Δ19 RNA. Culture supernatants from the transfected cells were collected at 5 days post-transfection (P0 samples) and inoculated into Vero E6 cells for amplification (P1 samples) and collected at 3 days post-inoculation (p.i.). RVFV Δ19 P1 samples were used for these studies and the 19 nt deletion was confirmed by sequencing of viral genomes obtained from P1 samples.

### Plaque assay

Plaque morphology of RVFV and RVFV Δ19 were determined in VeroE6 cells by plaque assay analysis used previously (Ikegami et al., 2006). Briefly, VeroE6 cells were inoculated with virus for 1 h at 37°C. After virus absorption, inoculum was removed and cells were overlaid with 1X modified eagle medium containing 0.6% Noble agar, 5% FBS, and 5% TPB. After 3 days of incubation, cells were stained with neutral red for 16 h at room temperature and plaques were visualized.

### Analysis of viral growth

Huh7 cells were infected with RVFV or RVFV Δ19 at a multiplicity of infection (MOI) of 1.0 or 0.01 at 37°C for 1 h. After virus absorption, cells were washed three times with PBS and growth medium was added. For the growth curve at an MOI of 1.0, culture supernatants were harvested at 0 h, 8 h, 12 h, 16 h, and 20 h p.i. For the growth curve at an MOI of 0.01, culture supernatants were harvested every day up to 4 days p.i. Virus titers were measured by plaque assay. The points in the growth curves are shown as mean +/- standard deviation from three independent experiments.

### Statistical analysis

Student’s t-test was used for growth curve analysis of RVFV and RVFV Δ19. Significant difference was defined as *p* < 0.05 (GraphPad Prism 9).

### Purification of virus particles

Culture supernatants harvested from virus-infected cells were purified as described previously (Terasaki et al., 2011). Briefly, supernatants were clarified by centrifugation at 3,000 rpm for 15 min. The clarified supernatant was layered on top of a discontinuous sucrose gradient and centrifuged for 3 h at 26,000 rpm at 4°C. The interface between 30 and 50% sucrose was collected and subjected to a second discontinuous sucrose gradient ultracentrifugation for 18 h at 26,000 rpm at 4°C. Virus was pelleted through a 20% sucrose cushion at 38,000 rpm for 2 h at 4°C. The pellets were suspended in Trizol reagent for RNA analysis.

### RNAse digestion

Huh7 cells were inoculated with RVFV at an MOI 3. After 8 h p.i., cells were mock-irradiated or irradiated with 254-nm-wavelength UV light at 250 mJ/cm^2^, followed by lysis of the UV-crosslinked cells using a high stringency SDS lysis buffer (0.5% SDS, 50 mM Tris-HCl [pH 6.8], 1 mM EDTA, 1 mM dithiothreitol [DTT]) and heating of the lysate to 65°C for 5 min. Radioimmunoprecipitation assay (RIPA) correction buffer (1.25% NP-40, 0.625% sodium deoxycholate, 62.5 mM Tris-HCl [pH 8.0], 2.25 mM EDTA, 187.5 mM NaCl) was then added to the lysates. Samples were passed through a QIAshredder spin column (Qiagen) twice to reduce viscosity. Lysates were then subjected to RNAse digestion using 30 µg RNAse A and 325 units of RNAse T1 for 3 h at 37°C. Total RNA was extracted with Trizol following the manufacturer’s instructions.

### CLIP-seq analysis

The protocol used by Sei and Conrad (2014) for the CLIP procedure was followed, with some modifications described previously (Tercero et al., 2021). RVFV-infected cells were irradiated with 254-nm-wavelength UV light at 250 mJ/cm^2^, followed by lysis of the UV-crosslinked cells using a high-stringency SDS lysis buffer (0.5% SDS, 50 mM Tris-HCl [pH 6.8], 1 mM EDTA, 1 mM DTT) and heating of the lysate to 65°C for 5 min. The high stringency lysis buffer condition disrupts the interaction of Gn with N and L proteins, thereby eliminating the possibility of indirect co-immunoprecipitation of viral RNAs with Gn protein through its interaction with N protein and/or L protein (Tercero et al., 2021). Subsequently, RIPA correction buffer was added to the lysates. To reduce viscosity, samples were passed through a QIAshredder spin column twice. Lysates were then subjected to RNAse digestion for 30 min at room temperature using an RNAse A/T1 mixture containing 30 µg RNAse A and 325 units of RNAse T1.

For immunoprecipitation, lysates were precleared using Dynabeads protein G, conjugated with unrelated mouse anti-H2K antibody (Tercero et al., 2021), for 15 min at 4°C with rotation. Subsequently, the precleared lysates were subjected to immunoprecipitation analysis using Dynabeads protein G, conjugated with mouse monoclonal anti-Gn antibody (R1-4D4) (Tercero et al., 2021) or anti-H2K antibody for 16 h at 4°C. The immunoprecipitated samples were washed 5 times with RIPA buffer containing a higher concentration of NaCl (high-salt RIPA buffer; 1% NP-40, 0.5% sodium deoxycholate, 0.1% SDS, 500 mM NaCl, 50 mM Tris-HCl [pH 8.0], 2 mM EDTA). Subsequently, the samples were resuspended in a high-salt RIPA buffer. For RNA extraction from intracellular and immunoprecipitated lysates, samples were resuspended in proteinase K solution (0.5 mg/ml of proteinase K, 0.5% SDS, 20 mM Tris-HCl [pH 7.5], 5 mM EDTA) and incubated at 37°C for 2 h. After proteinase K digestion, 3 M sodium acetate (NaOAc, pH 5.2) was added to the samples and RNA was extracted from the samples by phenol-chloroform/ethanol precipitation. The resulting RNA pellets were resuspended in sterile DNAse/RNAse-free H2O.

For preparation of RNA for CLIP-seq cDNA library construction, total RNA and immunoprecipitated RNA were subjected to dephosphorylation using 2 units of shrimp alkaline phosphatase for 30 min at 37°C and then 10 min at 65°C. After dephosphorylation, RNAs were phosphorylated using 10 units of T4 polynucleotide kinase for 30 min at 37°C and then 20 min at 65°C. After phosphorylation, RNAs were purified using miRNeasy Kit with RNeasy MiniElute Cleanup Kit (Qiagen) to obtain fragmented RNAs smaller than 200 nt. RNA was transferred to the Next Generation Sequencing Core facility at The University of Texas Medical Branch for adapter ligation and cDNA library construction. Library was sequenced using the NextSeq 550 Illumina system and bioinformatics analysis was performed by the Next Generation Sequencing Core facility. Reads were mapped to RVFV MP-12 genome (GenBank: DQ375404.1, DQ380208.1, and DQ380154.1) and visualized using Integrated Genome Viewer. Enriched regions (peaks) in the immunoprecipitated samples were normalized to enriched reads mapped to RVFV-infected total RNA.

### Strand-specific RT-qPCR

Reverse transcription (RT) of viral RNAs was performed using a strand-specific assay as described previously (Tercero et al., 2019). Briefly, cDNA synthesis of viral RNAs was performed by using the SuperScript III first-strand synthesis system (Invitrogen). Viral RNA was mixed with a 2 μM concentration of a strand-specific RT primer. To detect the antigenomic S RNA of RVFV Δ19, we used a strand-specific tagged RT primer that binds upstream of the deletion region. The mixture was incubated at 65°C for 5 min and then cooled to 4°C. After addition of the reaction buffer and enzyme mixture, cDNA synthesis was carried out by incubating the sample at 50°C for 55 min, with termination by heating at 85°C for 5 min, cooling to 4°C, and then treatment with RNAse H at 37°C for 20 min. The cDNAs were purified using an on-column PCR purification kit (Qiagen). The strand-specific real-time qPCR assays were conducted using SsoAdvanced Universal SYBR Green Supermix (Bio-Rad) and followed the established protocol as described previously (Tercero et al., 2019). Purified cDNA was added to the PCR master mix containing the nonviral tagged sequence as a forward primer and a viral strand-specific reverse primer. Thermocycling conditions were as follows: 95°C for 30 sec, 35 cycles of 95°C for 15 s and 60°C for 20 s, followed by melting-curve analysis. The assays were performed by using the CFX96 Touch real-time PCR detection system and analyzed using the provided software (Bio-Rad CFX Maestro).

### Northern blot analysis

Total RNAs were extracted from virus-infected cells using Trizol reagent (Invitrogen) according to the manufacturer’s protocol. The same amounts of total RNAs were subjected to Northern blot analysis using digoxigenin (DIG)-labeled RNA probes that specifically bind to the genomic L, M, S segments, and NSs mRNA (Ikegami et al., 2005b). A DIG-labeled IFN-β riboprobe was used for IFN-β mRNA detection (Narayanan et al., 2008). The RNAs were visualized using the DIG luminescent detection kit (Roche Applied Science), and images were analyzed using AlphaEaseFC software.

### Western blot analysis

Intracellular lysates were subjected to SDS-PAGE analysis using a 4 to 20% gel and transferred to a polyvinylidene difluoride (PVDF) membrane (Bio-Rad). For the detection of Gn, N and NSs proteins, mouse monoclonal anti-Gn antibody (R1-4D4), at a 1:3,000 dilution, rabbit polyclonal GST-N peptide antibody (R1-P1-GST-N) (Won et al., 2006), at a 1:3,000 dilution, and rabbit polyclonal NSs peptide antibody, at a 1:1,000 dilution (Ikegami et al., 2005b), were used as primary antibodies. A goat anti-mouse IgG HRP-linked antibody (Cell Signaling Technology), at a 1:10,000 dilution and goat anti-rabbit IgG HRP-linked antibody (Cell Signaling Technology), at a 1:10,000 dilution, were used as secondary antibodies, respectively. Chemiluminescence signals were detected with an ECL2 Western blotting kit (Thermo Scientific Pierce). Images were scanned, cropped, and assembled using AlphaEaseFC software.

## Results

### Identification of Gn-binding sites in the viral RNAs in RVFV-infected cells

**Figure 2:**
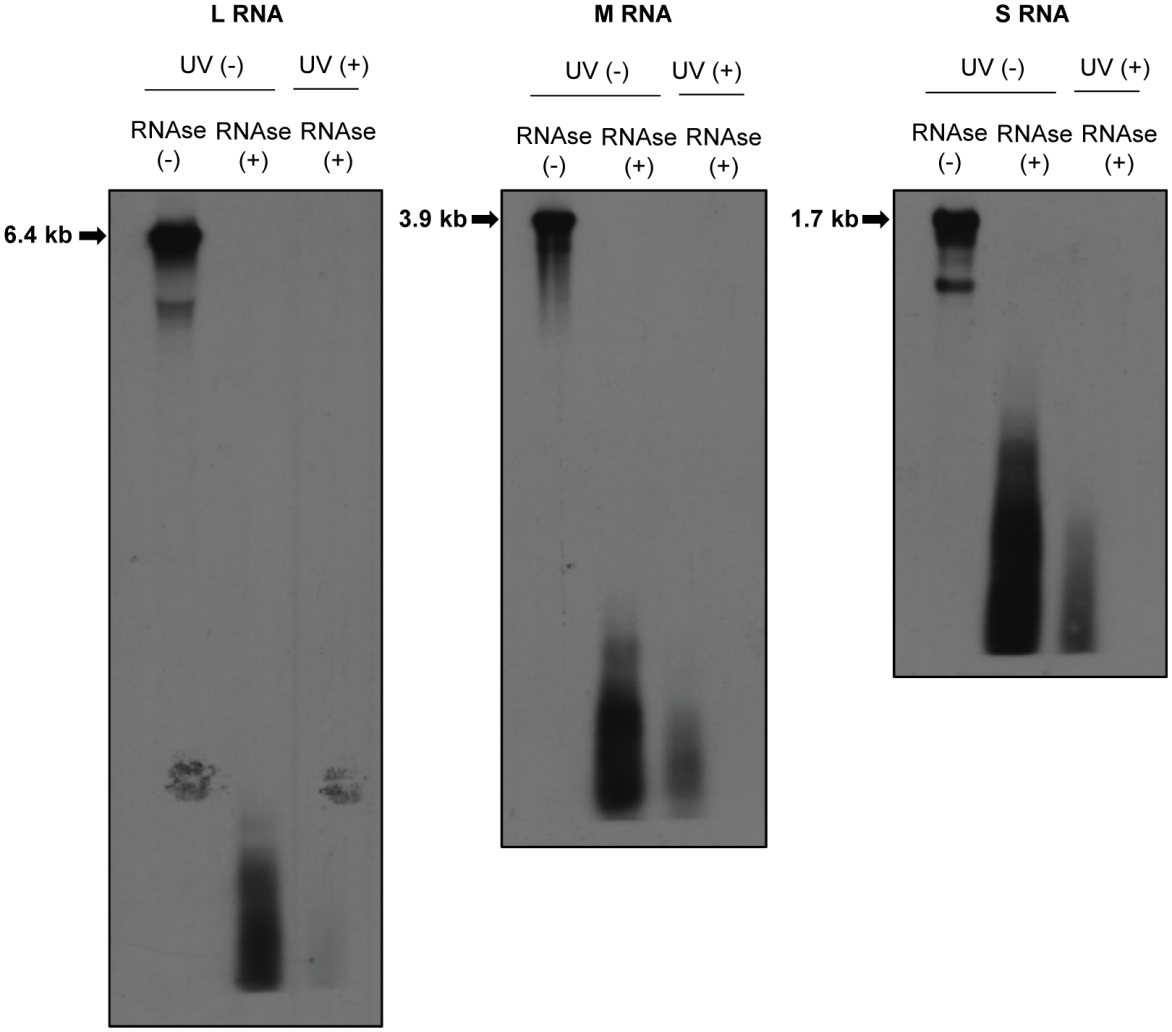
Susceptibilities of RVFV RNPs to RNAses. Huh7 cells were infected with RVFV at an MOI of 3 and mock-irradiated (UV -) or irradiated with UV light (UV +) at 8 h p.i. Cell extracts were prepared and treated with RNAse A/T1 mixture (RNAse +) for 3 h at 37˚C. After RNAse digestion, viral RNAs were extracted and subjected to Northern blot analysis using digoxigenin-labeled RNA probes, each of which specifically binds to genomic L (left panel), M (middle panel) or S RNA (right panel). Strong RNA signals indicated by arrows in the RNAse-untreated samples represent the viral genomic RNAs.

Previously, we reported the presence of a direct interaction between the envelope glycoprotein, Gn, and RVFV RNAs (Tercero et al., 2021). Furthermore, we observed a positive correlation between the ability of Gn to bind viral RNAs and the packaging ability of RVFV RNA segments into virus particles (Tercero et al., 2021). To gain an insight into the mechanism of RNA packaging in RVFV, we sought to further characterize this interaction by identifying regions in viral RNAs that directly interact with Gn using CLIP-seq analysis. This analysis requires the fragmentation of viral RNAs in RVFV-infected cells by partial RNAse digestion to generate fragments with sizes ideal for subsequent high-throughput sequencing. However, a past study suggested that RVFV RNPs are resistant to RNAse treatment (Raymond et al., 2010), which would impact the feasibility of conducting CLIP-seq analysis. To determine the susceptibility of RVFV RNAs in RNP complexes to RNAse-digestion, we treated the lysate of RVFV-infected cells with a mixture of RNAse A/T1, extracted RNAs after the RNAse treatment, and analyzed the integrity of the genomic RNAs by Northern blot analysis. We detected a smear of small-sized viral RNA signals, but not full-length genomic RNAs, in the RNAse-treated samples (**Figure 2**), demonstrating the susceptibility of viral RNAs, including those in the viral RNPs, to RNAse treatment.

**Figure 3:**
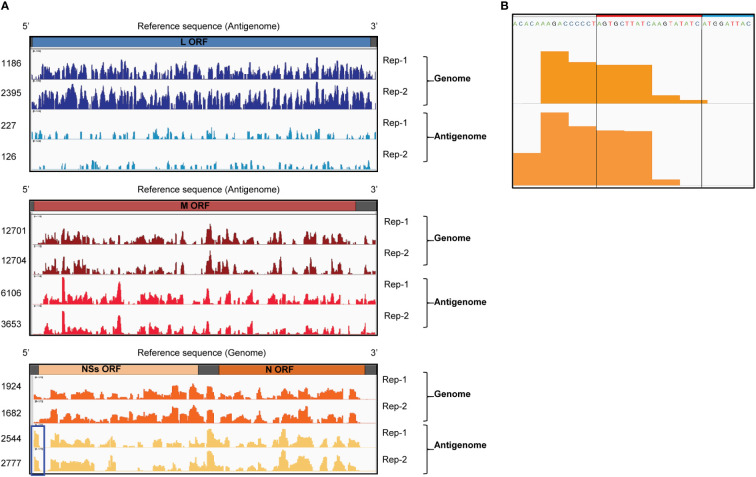
CLIP-seq analysis of Gn-binding sites in RVFV RNA segments. **(A)** CLIP-seq read coverage plots of RVFV genomic and antigenomic RNA segments bound by Gn: L RNA (top plot), M RNA (middle plot), and S RNA (bottom plot). Read coverage plots of immunoprecipitated RNAs were generated after normalization with reads obtained from intracellular RNA (input control). Read coverages of each viral RNA are visualized by Integrative Genomics Viewer and read values are indicated on the left side of the coverage plots. Two independent biological replicates are plotted and designated by Rep-1 and Rep-2. Blue box indicates a prominent peak within the 3’ NCR of antigenomic S RNA. ORF = open reading frame **(B)** The sequence and CLIP-seq read coverage plots (orange-colored areas) of the 3’ NCR of the antigenomic S RNA (Rep-1 and Rep-2). The 19 nt region, which is deleted in RVFV Δ19, is indicated by a red box and a portion of the NSs ORF is shown by a blue box.

We performed CLIP-seq analysis using two independent samples. Briefly, the UV-irradiated and RNAse-treated extracts from RVFV-infected cells were subjected to co-immunoprecipitation analysis using an unrelated anti-H2K antibody (Tercero et al., 2021) (control group) or anti-RVFV Gn antibody (experimental group). Fragmented total RNAs and co-immunoprecipitated RNAs were collected and prepared for cDNA library construction. The cDNAs were subjected to next generation sequencing followed by bioinformatics analysis. Subsequently, read coverage plots of the co-immunoprecipitated RNAs were constructed. The control group showed a low number of reads at negligible levels, whereas the experimental group showed a significant number of reads that mapped to viral RNAs. The read coverage plots of the genomic and antigenomic viral RNAs, which were normalized by subtracting the reads of intracellular viral RNAs (input control) from the reads of the experimental group, were visualized by Integrative Genomics Viewer and are shown in **Figure 3A**. The pronounced peaks represent the regions with high read density. The read coverage plots of two independent experiments were similar for most of the viral RNAs, demonstrating good reproducibility. The differences in the read coverage plots of antigenomic L RNA between the two independent experiments were larger than that for other viral RNAs, potentially due to low read coverage for this RNA. The proportion of reads that mapped to genomic L and M RNAs were substantially higher than that to their antigenomic counterparts. In addition, the number of reads that mapped to genomic and antigenomic S RNAs were similar, with a slightly higher number of reads mapping to antigenomic S RNA. These data correlated with our previous studies that demonstrated the efficient binding of Gn to the genomic L, M and S RNAs and antigenomic S RNA (Tercero et al., 2021). The presence of similar read coverage plots for a given viral RNA segment in two independent experiments strongly suggested that the prominent regions of sequencing reads in the plots represent sites in viral RNAs that preferentially interact with Gn. Hence, our CLIP-seq analysis indicated direct binding of Gn to many different regions of the genomic and antigenomic RVFV RNAs.

No prominent sequence coverage that aligns with the minimal NCRs of most of the viral RNAs were detected, except for the 3’ NCR of the antigenomic S RNA, which had a prominent number of sequencing reads (highlighted by a blue box in **Figure 3A**). **Figure 3B** shows the sequence and read coverage plots of the 3’ terminal region of the antigenomic S RNA. Within the 34 nt-long 3’ NCR, the terminal 15 nt is essential for RNA replication and packaging of the genomic RNA of the S RNA-derived minigenome RNA (Murakami et al., 2012). The region corresponding to 5-25 nt from the 3’ NCR of the antigenomic S RNA showed a high read density in two independent experiments.

### Rescue of a RVFV mutant carrying a deletion at the 3’ NCR of antigenomic S RNA

**Figure 4:**
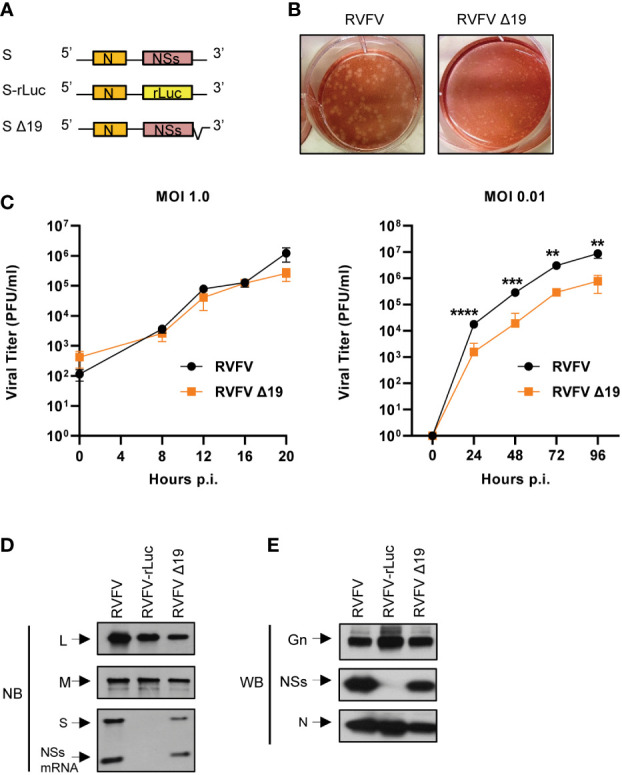
Rescue and characterization of RVFV Δ19. **(A)** Schematic diagram of S RNA and S RNA mutants in the antigenomic sense. S-rLuc RNA contains the rLuc ORF in place of the NSs ORF. S Δ19 RNA contains a deletion of 19 nt in the 3’-terminal NCR of the antigenomic S RNA. **(B)** Plaque morphologies of RVFV and RVFV Δ19 in Vero E6 cells. **(C)** Growth curves of RVFV and RVFV Δ19 in Huh7 cells. Huh7 cells were infected with RVFV or RVFV Δ19 at an MOI of 1.0 (left panel) or an MOI of 0.01 (right panel). Culture supernatants were collected at the indicated time points and the virus titers were determined in Vero E6 cells by plaque assay analysis. The data were obtained from three independent experiments. The values are mean titers and error bars indicate standard deviation. **P < 0.01, ***P < 0.001, ****P < 0.0001. **(D)** Accumulation of viral RNAs in infected cells. Huh7 cells were infected with RVFV, RVFV-rLuc, or RVFV Δ19 at an MOI of 3. Total intracellular RNAs were extracted at 8 h p.i and equal amounts of intracellular RNAs were analyzed by Northern blot (NB) using digoxigenin-labeled RNA probes that specifically bind to the respective genomic RNA segments. The RNA probe to detect genomic S binds within the NSs ORF resulting in detection of full-length S RNA as well as NSs mRNA. **(E)** Accumulation of viral proteins in infected cells. Huh7 cells were infected with RVFV, RVFV-rLuc or RVFV Δ19 at an MOI of 3. Cell lysates were collected at 8 h p.i. using the same amount of sample buffer and subjected to Western blot (WB) analysis using anti-Gn antibody (top panel), anti-NSs antibody (middle panel) and anti-N antibody (bottom panel).

To know the biological significance of the prominent Gn-binding site within the 3’ NCR of antigenomic S RNA, we rescued a RVFV mutant (RVFV Δ19) that has a 19 nt deletion in the 3’-NCR of the antigenomic S RNA using a reverse genetics system (Ikegami et al., 2006) (**Figures 3B**, **4A**). RVFV Δ19 retained the 3’ terminal 15 nt of NCR and lacked about 50% of the region that showed prominent number of reads within the 3’ NCR of antigenomic S RNA in CLIP-seq analysis. RVFV Δ19 formed smaller plaques than RVFV in Vero E6 cells (**Figure 4B**). RVFV Δ19 replicated to comparable titers to RVFV in Huh7 cells at a high MOI (**Figure 4C**, left panel), but replicated less efficiently than RVFV at a low MOI (**Figure 4C**, right panel).

As the 19 nt deletion is located upstream of the NSs mRNA start codon (**Figure 3B**), we examined whether NSs expression occurs in RVFV Δ19-infected cells. We used RVFV and RVFV-rLuc, the latter of which encodes rLuc gene in place of NSs gene (**Figure 4A**) (Ikegami et al., 2006), as a positive control and a negative control, respectively. Like RVFV-infected cells, accumulation of the genomic L, M and S RNAs and NSs mRNA occurred in RVFV Δ19-infected cells (**Figure 4D**). The absence of the genomic S RNA and NSs mRNA in RVFV-rLuc-infected cells was due to the use of the probe that binds to the NSs gene. Accumulation of Gn, N and NSs proteins also occurred in RVFV Δ19-infected cells (**Figure 4E**). As expected, accumulation of NSs did not occur in RVFV-rLuc-infected cells (**Figure 4E**). These data demonstrated that RVFV Δ19 did not replicate as efficiently as RVFV after low MOI infection in Huh7 cells, although NSs mRNA synthesis still occurred from the antigenomic S RNA of RVFV Δ19.

### Inefficient packaging of antigenomic S RNA into RVFVΔ19, lacking the Gn-binding site in the 3’ NCR of antigenomic S RNA

**Figure 5:**
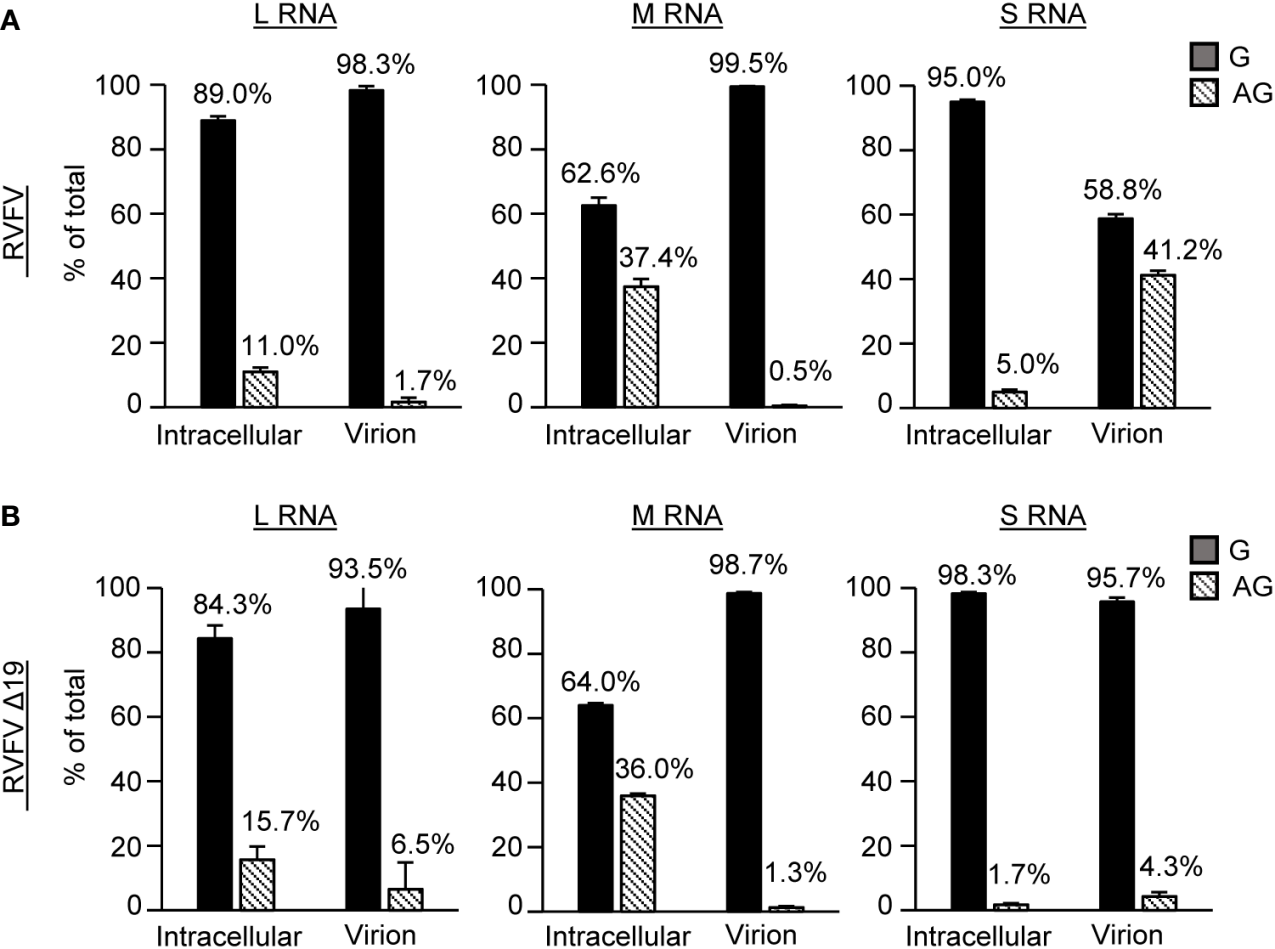
Profile of viral RNA segments packaged into purified RVFV and RVFV Δ19 particles. Proportions of genomic and antigenomic RVFV RNA segments in **(A)** RVFV and **(B)** RVFV Δ19-infected cells and purified virus particles. Huh7 cells were infected with RVFV or RVFV Δ19 at an MOI of 2. Intracellular lysates and culture supernatants were collected at 8 h p.i. Virus particles released in the culture supernatant were purified by sucrose gradient ultracentrifugation. The copy numbers of genomic (G) and antigenomic (AG) L, M, S RNAs in intracellular lysate and in purified virions were determined by strand-specific RT-qPCR. The proportions of genomic and antigenomic RNAs in virus-infected cells and in purified virions are represented as a percentage of total viral RNA for each segment. Data represent the means of biological triplicates, and error bars indicate the standard deviations.

To determine if the 19 nt region in the 3’ NCR of antigenomic S RNA played a role in its efficient packaging into virus particles, we examined the proportion of the genomic and antigenomic viral RNAs in RVFV Δ19-infected cells and in purified RVFV Δ19 particles and compared the data to that obtained from RVFV. As reported previously (Tercero et al., 2021), the proportion of the antigenomic S RNA in RVFV virions was markedly higher relative to its abundance in infected cells and the relative proportion of antigenomic S RNA in virus particles was prominently higher than that of antigenomic L and M RNAs (**Figure 5**). In contrast, the proportion of the antigenomic S RNA in RVFV Δ19 virions relative to its abundance in infected cells was markedly lower than that in RVFV-infected cells, demonstrating that antigenomic S RNA is inefficiently packaged into RVFV Δ19. These data showed that the prominent Gn-binding site in the 3’ NCR of antigenomic S RNA plays a critical role in the efficient packaging of antigenomic S RNA into virus particles.

### Inefficient packaging of antigenomic S RNA into RVFV particles results in the early induction of IFN-β mRNA after infection

**Figure 6:**
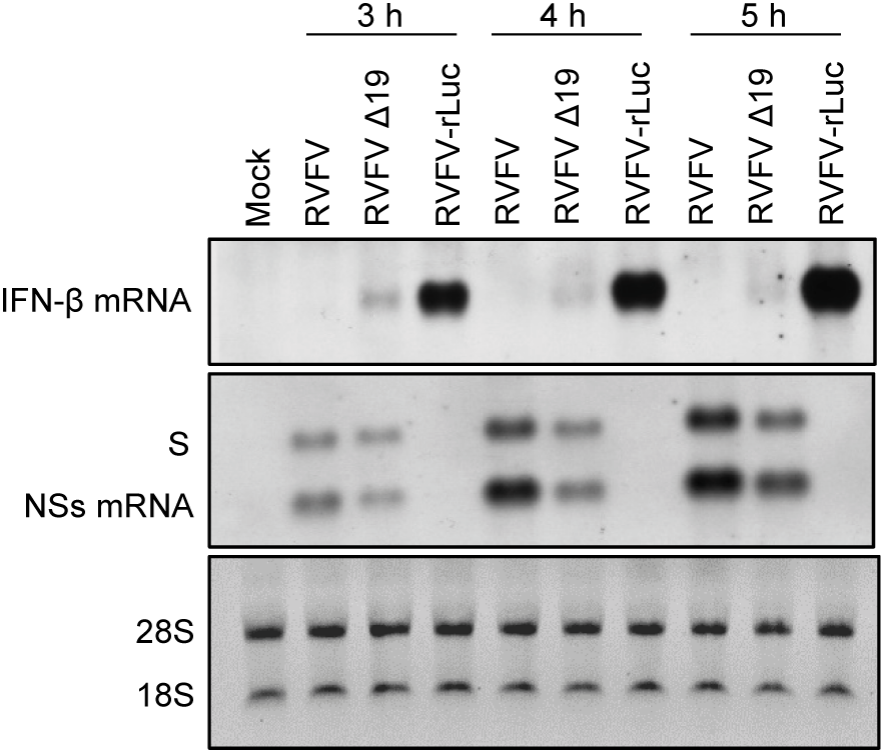
Accumulation of the genomic S RNA, NSs mRNA and IFN-β mRNA in MRC-5 cells infected with RVFV, RVFV Δ19, or RVFV-rLuc. MRC-5 cells were infected with RVFV, RVFV Δ19, or RVFV-rLuc at an MOI of 3. Total RNAs were collected at various time p.i. as indicated and equal amounts of intracellular RNAs were applied to Northern blot analyses. IFN-β mRNA was detected by using the IFN-β mRNA-specific RNA probe (top panel). Genomic S RNA and NSs mRNA were detected by using a specific RNA probe that binds within the NSs ORF (middle panel). The 28S and 18S ribosomal RNAs (internal loading control) were stained with ethidium bromide, following the separation of the same RNA samples by agarose gel electrophoresis (bottom panel).

Our past study indicated that the incoming antigenomic S RNA in RVFV particles serves as the template for NSs mRNA synthesis, leading to the synthesis of NSs protein immediately after infection (Ikegami et al., 2005b). Early expression of NSs after infection would be important for robust RVFV replication, as the incoming viral RNPs can trigger type I IFN induction (Weber et al., 2013) and NSs suppresses host innate immune responses, including type I IFN induction by inhibiting IFN-β mRNA transcription (Le May et al., 2008). To further establish the importance of the efficient packaging of antigenomic S RNA for the suppression of the early induction of type I IFN after infection, we infected type I IFN-competent MRC-5 cells (Terasaki et al., 2011) with RVFV, RVFV Δ19 or RVFV-rLuc and examined the induction of IFN-β mRNA from 3 to 5 h p.i. (**Figure 6**). RVFV replication did not induce IFN-β mRNA, whereas RVFV-rLuc replication induced high levels of IFN-β mRNA, as reported previously (Terasaki et al., 2011). Notably, we detected IFN-β mRNA, the amount of which was highest at 3 h p.i., in RVFV Δ19-infected cells (**Figure 6**). Northern blot analysis using an RNA probe that binds to the NSs gene showed the accumulation of NSs mRNA in RVFV Δ19-infected cells and in RVFV-infected cells during the early timepoints after infection (**Figure 6**). Specifically, at 3 h p.i. the amount of NSs mRNA was lower in RVFV Δ19-infected cells compared to that in RVFV-infected cells, while the amount of genomic S RNA was comparable between these cells (**Figure 6**). It is unlikely that the lower accumulation of NSs mRNA in RVFV Δ19-infected cells at 3 h p.i. is due to the reduced transcriptional activity from the antigenomic S RNA of RVFV Δ19, as the relative ratio of NSs mRNA to the genomic S RNA in RVFV Δ19-infected cells and in RVFV-infected cells were similar at 5 h p.i. (**Figure 6**). Rather, these data suggest that the presence of lower amounts of incoming antigenomic S RNA, which serves as the template of NSs mRNA, resulted in the less efficient accumulation of NSs mRNA immediately after RVFV Δ19 infection. Taken together, these data indicate that the inefficient packaging of antigenomic S RNA into RVFV Δ19 particles led to a decreased accumulation of NSs mRNA during the early timepoints after infection with RVFV Δ19. In RVFV-rLuc-infected cells, IFN-β mRNA expression increased between 3 and 5 h p.i. compared to RVFV-infected cells (**Figure 6**), whereas the IFN-β mRNA expression decreased at 4 and 5 h p.i. in RVFV Δ19-infected cells. These findings suggest that the NSs expression increased by 5 h p.i. in RVFV Δ19-infected cells (**Figure 6**), due to the subsequent accumulation of NSs mRNA following the replication of S RNA, leading to the suppression of IFN-β mRNA synthesis later in infection.

## Discussion

The present study represents a continuation of our research clarifying the mechanism of viral RNA packaging in RVFV (Ikegami et al., 2005b; Terasaki et al., 2011; Murakami et al., 2012; Tercero et al., 2021). To test the feasibility of the CLIP-seq analysis, we examined the susceptibility of intracellular viral RNAs, including those in the viral RNPs, to RNAse treatment and revealed the susceptibility of viral RNAs to RNAse treatment (**Figure 2**). Our data were consistent with a recent study showing the susceptibility of intracellular RVFV RNAs to RNAses (Hayashi et al., 2021). For the interpretation of the CLIP-seq data, it is important to note that the antigenomic L RNA and antigenomic M RNA share the same sequences with L mRNA and M mRNA, respectively, except that these mRNAs lack a portion of the 3’-end region of their corresponding antigenomic RNAs (Ikegami et al., 2007) (see **Figure 1**). Similarly, for the S segment, the NSs mRNA and N mRNA share the same sequence with a portion of the genomic S RNA and antigenomic S RNA, respectively (Ikegami et al., 2007) (see **Figure 1**). We cannot exclude the possibility that the CLIP-seq reads, which mapped to these overlapping regions among RVFV RNA species, could also include those that are generated from viral mRNAs. However, we speculate that as viral mRNAs are most probably not packaged into RVFV particles, Gn does not bind to viral mRNAs, and that the CLIP-seq read coverage plot predominantly represent binding sites for Gn on viral genomic and antigenomic RNAs. To our knowledge, our CLIP-seq analysis data, which showed the direct binding of Gn to many different regions of the genomic as well as antigenomic RVFV RNAs, represents the first study that demonstrates the direct binding of a viral envelope glycoprotein to distinct viral RNA regions in an enveloped RNA virus, including those in the order Bunyavirales.

Because the presence of the minimal NCRs is sufficient for minigenome RNA replication and packaging of the genomic sense of minigenome RNAs into RVFV particles (Murakami et al., 2012), our expectation was to detect the presence of prominent Gn-binding sites within the minimal NCRs, which serve as packaging signals to initiate Gn-viral RNA interaction, resulting in the incorporation of viral RNAs into RVFV particles. In contrast to our expectation, CLIP-seq analysis showed that most of the minimal NCRs lacked prominent Gn-binding sites, except for a site within the 3’ NCR of the antigenomic S RNA. These data suggest that the efficient packaging of genomic RNAs occurs in the absence of a direct interaction between Gn and minimal NCRs of genomic RNAs. Although the region encompassing the NSs ORF of the antigenomic S RNA included several Gn-binding sites (**Figure 3**), they are probably not essential for the efficient packaging of antigenomic S RNA, because the antigenomic S RNA of RVFV-rLuc, lacking the NSs ORF, is efficiently incorporated into virus particles (Tercero et al., 2021). Furthermore, antigenomic S RNA was not efficiently packaged into RVFV Δ19 that had an intact NSs ORF (**Figure 5**). These data imply that most of the Gn-binding sites in the viral RNAs, except for the one within the 3’ NCR of antigenomic S RNA, do not play a role in the initiation of the interaction between Gn and viral RNPs. It is conceivable to speculate that the interaction between Gn and viral RNPs is triggered by the binding to Gn to L or N protein in the genomic viral RNPs (Piper et al., 2011; Murakami et al., 2012; Hayashi et al., 2021; Tercero et al., 2021). Subsequently, this interaction is stabilized by the binding of Gn to various regions of viral RNAs (**Figure 3**), ensuring the efficient packaging of viral RNPs into virus particles.

Analysis of RVFV Δ19 suggested that the interaction between Gn and the prominent Gn-binding site within the 3’ NCR of antigenomic S RNA facilitated the efficient packaging of antigenomic S RNA. This prominent Gn-binding site could serve as a packaging signal for antigenomic S RNA, which is recognized by Gn to initiate its interaction with antigenomic S RNP. The binding of Gn to L or N protein in the antigenomic S RNP could also be the trigger to establish its interaction with antigenomic S RNP. These two potential mechanisms of recognition and initiation of the interaction of Gn with antigenomic S RNPs may not be mutually exclusive and could work in concert to form a stable packaging-competent complex, resulting in the efficient packaging of antigenomic S RNA into virus particles. Testing this hypothesis would be meaningful to gain further insight into the mechanism of viral RNA packaging in RVFV. We also wonder whether this putative packaging signal is a transferable packaging element that has biological activity when introduced into any other RVFV RNA segment. If so, a RVFV mutant carrying this packaging signal in another viral RNA segment(s) would exhibit an altered profile of viral RNA packaging, be less virulent and potentially, could be developed as a safer, live-attenuated RVFV vaccine.

RVFV Δ19, lacking a part of this prominent Gn-binding site in antigenomic S RNA, was competent for S RNA replication and transcription, as S RNA replication and NSs mRNA transcription occurred in RVFV Δ19-infected cells (**Figures 4D**, **6**). Robust accumulation of N protein (**Figure 4E**) also suggested the efficient synthesis of N mRNA in RVFV Δ19-infected cells. However, we observed a slightly reduced accumulation of S RNA and NSs mRNA in RVFV Δ19-infected cells (**Figures 4D**, **6**), which suggested that the 19 nt region in S RNA was important for optimal S RNA replication and transcription. Although it is possible that this could broadly affect the overall packaging efficiency of RVFV RNAs, the predominant negative impact on RNA packaging efficiency was observed only for antigenomic S RNA (**Figure 5**). Hence, our data suggests that the primary biological function of the 19 nt region in the 3’ NCR of antigenomic S RNA was to drive the efficient packaging of antigenomic S RNA into virus particles. Also, RVFV Δ19 replicated less efficiently than RVFV in Huh7 cells after low MOI infection (**Figure 4C**), demonstrating that the presence of the 19 nt in the 3’ NCR of the antigenomic S RNA is required for optimal RVFV replication.

We showed the early accumulation of IFN-β mRNA in RVFV Δ19-infected cells, but not in RVFV-infected cells (**Figure 6**). The accumulation of NSs mRNA was slightly higher in RVFV-infected cells than in RVFV Δ19-infected cells at 3 h p.i., although the difference was small (**Figure 6**). These data imply the presence of a mechanism that ensures the optimal packaging of antigenomic S RNA into RVFV, which is sufficient to suppress the early induction of IFN-β mRNA expression after infection. The data shown in this study and our previous study (Ikegami et al., 2005b) have firmly established the importance of efficient packaging of antigenomic S RNA for the inhibition of the early induction of IFN-β mRNA after RVFV infection.

## Data availability statement

The datasets presented in this study can be found in online repositories. The names of the repository/repositories and accession number(s) can be found below: https://www.ncbi.nlm.nih.gov/bioproject/PRJNA925541/.

## Author contributions

BT and SM conceptualized the study. BT, KT and KN performed experiments. BT, KT, KN and SM analyzed the data. BT and SM wrote the manuscript with contributions from KT and KN. All authors contributed to the article and approved the submitted version.
